# Patient Interaction Phenotypes With an Automated Remote Hypertension Monitoring Program and Their Association With Blood Pressure Control: Observational Study

**DOI:** 10.2196/22493

**Published:** 2020-12-03

**Authors:** Anahita Davoudi, Natalie S Lee, Corey Chivers, Timothy Delaney, Elizabeth L Asch, Catherine Reitz, Shivan J Mehta, Krisda H Chaiyachati, Danielle L Mowery

**Affiliations:** 1 Department of Biostatistics, Epidemiology & Informatics University of Pennsylvania Philadelphia, PA United States; 2 National Clinician Scholars Program University of Pennsylvania Philadelphia, PA United States; 3 Leonard Davis Institute of Health Economics University of Pennsylvania Philadelphia, PA United States; 4 Corporal Michael J Crescenz Veterans Affairs Medical Center Philadelphia, PA United States; 5 Penn Medicine Predictive Healthcare University of Pennsylvania Health System Philadelphia, PA United States; 6 Center for Healthcare Innovation University of Pennsylvania Philadelphia, PA United States; 7 Department of Medicine Perelman School of Medicine University of Pennsylvania Philadelphia, PA United States; 8 Institute for Biomedical Informatics University of Pennsylvania Philadelphia, PA United States

**Keywords:** text messaging, hypertension, telemedicine, cluster analysis

## Abstract

**Background:**

Automated texting platforms have emerged as a tool to facilitate communication between patients and health care providers with variable effects on achieving target blood pressure (BP). Understanding differences in the way patients interact with these communication platforms can inform their use and design for hypertension management.

**Objective:**

Our primary aim was to explore the unique phenotypes of patient interactions with an automated text messaging platform for BP monitoring. Our secondary aim was to estimate associations between interaction phenotypes and BP control.

**Methods:**

This study was a secondary analysis of data from a randomized controlled trial for adults with poorly controlled hypertension. A total of 201 patients with established primary care were assigned to the automated texting platform; messages exchanged throughout the 4-month program were analyzed. We used the *k*-means clustering algorithm to characterize two different interaction phenotypes: program conformity and engagement style. First, we identified unique clusters signifying differences in program conformity based on the frequency over time of error alerts, which were generated to patients when they deviated from the requested text message format (eg, ###/## for BP). Second, we explored overall engagement styles, defined by error alerts and responsiveness to text prompts, unprompted messages, and word count averages. Finally, we applied the chi-square test to identify associations between each interaction phenotype and achieving the target BP.

**Results:**

We observed 3 categories of program conformity based on their frequency of error alerts: those who immediately and consistently submitted texts without system errors (perfect users, 51/201), those who did so after an initial learning period (adaptive users, 66/201), and those who consistently submitted messages generating errors to the platform (nonadaptive users, 38/201). Next, we observed 3 categories of engagement style: the enthusiast, who tended to submit unprompted messages with high word counts (17/155); the student, who inconsistently engaged (35/155); and the minimalist, who engaged only when prompted (103/155). Of all 6 phenotypes, we observed a statistically significant association between patients demonstrating the minimalist communication style (high adherence, few unprompted messages, limited information sharing) and achieving target BP (*P*<.001).

**Conclusions:**

We identified unique interaction phenotypes among patients engaging with an automated text message platform for remote BP monitoring. Only the minimalist communication style was associated with achieving target BP. Identifying and understanding interaction phenotypes may be useful for tailoring future automated texting interactions and designing future interventions to achieve better BP control.

## Introduction

Hypertension is a salient risk factor for heart disease and stroke [1]. Home blood pressure (BP) monitoring has long been accepted as a valid strategy for effective hypertension management [2]. Patients adhere to telemonitoring programs and use this technology for chronic conditions such as hypertension regardless of their nationality, socioeconomic status, or age [3]. In light of recent Centers for Medicare & Medicaid Services codes that reimburse remote monitoring of physiologic parameters such as BP and a shift toward greater remote monitoring because of the COVID-19 pandemic, remote BP monitoring is poised to become an increasingly common strategy for hypertension management.

An estimated 96% of Americans own mobile phones with text messaging capabilities [4], and mobile phone-based interventions are increasingly popular for remote disease management. Texting might be the best phone-based modality for disease management, as general patient populations are less likely to use smartphones, tablets, and health-related apps relative to texting [5]. Text messaging is an appealing platform for remote management given its accessibility and low costs, which may help reduce disparities in health care [6].

Automated texting in particular has been leveraged nationally and globally for remote hypertension management, with high patient engagement and satisfaction among low-income and underserved populations [7,8]. Automated texting interventions can double the odds of medication adherence for chronic conditions, regardless of whether texts are unidirectional or interactive [9]. Automated interactive texting may be an especially effective method of engaging patients in BP self-monitoring [10,11]. However, the impact of automated texting on BP targets is less clear, with mixed evidence about its effectiveness. Randomized trials suggest no effect of automated texting on achieving target BP [12,13], while other evidence suggests varying degrees of benefit [14,15].

These heterogeneous findings may be explained by different ways patients engage with texting platforms (behavioral phenotypes) [16]. Outside of changing the frequency of reminders or word choices, automated interventions using texting platforms have generally taken a one-size-fits-all approach to how patients should engage with the platform. However, patients likely differ in their interactions with and responses to automated texts. These variations are reflective of how individuals converse via text messaging (eg, quantity and quality of texting) and may correlate with how they engage in treatment, ultimately affecting clinical outcomes from automated texting interventions. We hypothesized that discrete behavioral phenotypes existed among patients engaging with clinical automated texting programs and that some phenotypes were likely to achieve targeted clinical outcomes (eg, controlled BP). Identifying and understanding the various ways patients engage with automated texting (phenotypes) would provide greater insights for targeting specific behaviors and tailoring interventions to improve hypertension control. For example, phenotypes associated with poor BP control may require more intensive texting approaches or more in-person care.

The purpose of this study was to identify and describe unique phenotypes of patient interactions with automated texting for remote BP management and estimate associations between interaction phenotypes and achieving a target BP.

## Methods

### Original Randomized Trial Description

In this University of Pennsylvania institute review board–approved study (828417, 834667), we retrospectively studied text messages and clinical data from adult patients in a randomized controlled trial (ClinicalTrials.gov NCT03416283) who were receiving automated text-based reminders for hypertension management. This trial’s primary aim was to leverage automated text messaging for remote BP monitoring with or without social support to improve hypertension control over a 4-month program. Our study is a posttrial analysis that was independent of the study aims. Eligible participants were aged 18 to 75 years and had had at least 2 office visits at the Penn Family Care practice in Philadelphia, PA, with at least 2 office visit BP readings above goal (140/90 mm Hg) within 24 months prior to enrolling in the trial.

### Study Context: Remote Monitoring With and Without Social Support Groups Trial

In the original study, scheduled automated text messages were used to (1) monitor each patient’s BP measurements over time, (2) provide intermittent encouragement for engaging behavior, and (3) monitor BP medication adherence. Texts were sent through Way to Health, a Health Insurance Portability and Accountability Act–compliant, bidirectional, automated text communication platform used to engage with patients for research studies and clinical care delivery [17,18]. Patients in the original study received a variety of outbound texts including information about the study and upcoming in-person study visits. The three most frequent outbound messages include:

- Blood pressure prompts: “What is your blood pressure today (Ex. 120/80)?”
- Feedback: “You submitted X out of 3 BP measurements this week, great job!”
- Medication adherence: “How many days did you take your blood pressure medication(s) in the past 7 days? (Please input a single number: 0-7)”

**Figure 1:**
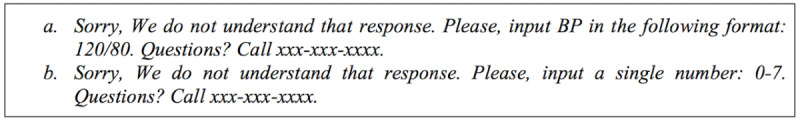
Error messages: (a) blood pressure and (b) medication adherence.

The software was programmed to receive responses in a prespecified, structured format. For example, the reminder “What is your blood pressure today?” accepted a text response formatted as ###/## with few allowable variations, and the medication adherence prompt required a single number between 0 and 7. When patients submitted a correctly formatted text in response to these prompts, they received an automated confirmation text. However, as this was a text-based program, patients were not prevented from submitting text messages of any length in any form, at any time. Those message that did not conform to the requested text format triggered automated error messages. For example, when the text was not submitted in the expected format, patients received an automated error message with instructions to resubmit a response in the correct format. Not all formatting errors triggered an alert. For example, text messages sent immediately following an error message or messages that were not in direct response to an automated prompt (ie, were unprompted) did not generate these alerts. Examples of the reminder-specific error messages are shown in Figure 1.

There were two intervention arms. The first arm received automated text messages as described above. The second received the same automated text messages and identified a person in their social support network (eg, a family member or friend) who also received text-based reports about the study participant’s performance in the program. The control arm received no text messages. Randomization was in a 2:2:1 ratio for the two intervention arms and a control group, with 201 patients randomized to the two text intervention arms. In the original trial, 101 patients were enrolled in the remote monitoring only group (RM) and 100 in the remote monitoring + social support group (RM+SS). BP readings that were consistently out of range were escalated to the clinical care team via the electronic health record.

### Characterizing Interaction Phenotypes

For our posttrial analysis, we focused on the 201 patients in the intervention arms to identify phenotypes of patient interactions with the automated text messaging system. We prespecified two categories of interaction phenotypes, program conformity and engagement style.

### Program Conformity

Program conformity refers to the ways that users complied with the platform’s requirements for text message communication. We used *k*-means clustering, an unsupervised learning method and data-driven approach to classify subgroups of observations within a dataset into *k* clusters based on each observation’s proximity to a cluster mean or centroid [19]. Clusters were formed based on variations in error messages over time signifying program conformity. To capture the temporal variation of the error messages, we tallied the number of error messages at 20-day intervals and divided each 20-day total by the total number of messages by the patient in that time frame. Each patient must have submitted at least 1 message to be counted within a time frame and consistently engaged by submitting messages throughout all time frames to be included in the analysis. These features were fed into the *k*-means clustering algorithm to draw 3 clusters from the data signifying different program conformity user categories.

### Engagement Style

Engagement style is characterized by observable patterns of engagement with the overall BP monitoring program. Engagement style patterns were based on a broader family of variables that were prespecified, including the following:

- Proportion of responses to 48 total BP prompts signifying program compliance (BP reporting adherence)
- Average word count per message signifying verbosity (word average), excluding numbers like BP measurements
- Proportion of inbound messages that were not in response to an automated text message prompt (unprompted messages)
- Proportion of patient-submitted messages that triggered an error alert (error rate)

All proportions and word count averages were standardized to a scale between 0 and 1 for the analysis. Again, using the *k*-means clustering algorithm, we identified 3 distinct clusters of engagement styles. To further establish a qualitative understanding of each cluster, we identified the intents—the intended subject of communication (eg, greetings, pleasantry, medication, question, etc)—of all inbound messages from patients that were exceptions to the expected structured text response. Each message was annotated by two members of the research team, applying a common annotation codebook for intents and allowing for multiple intents per message. Discrepancies between the two reviewers were resolved through consensus review. We also described each cluster according to participant age, sex, and race and intervention study arm. Only patients who completed the intervention and had their data entered into the Research Electronic Data Capture database were included in the analysis.

### Identifying Associations Between Interaction Phenotypes With Blood Pressure Outcomes

**Figure 2:**
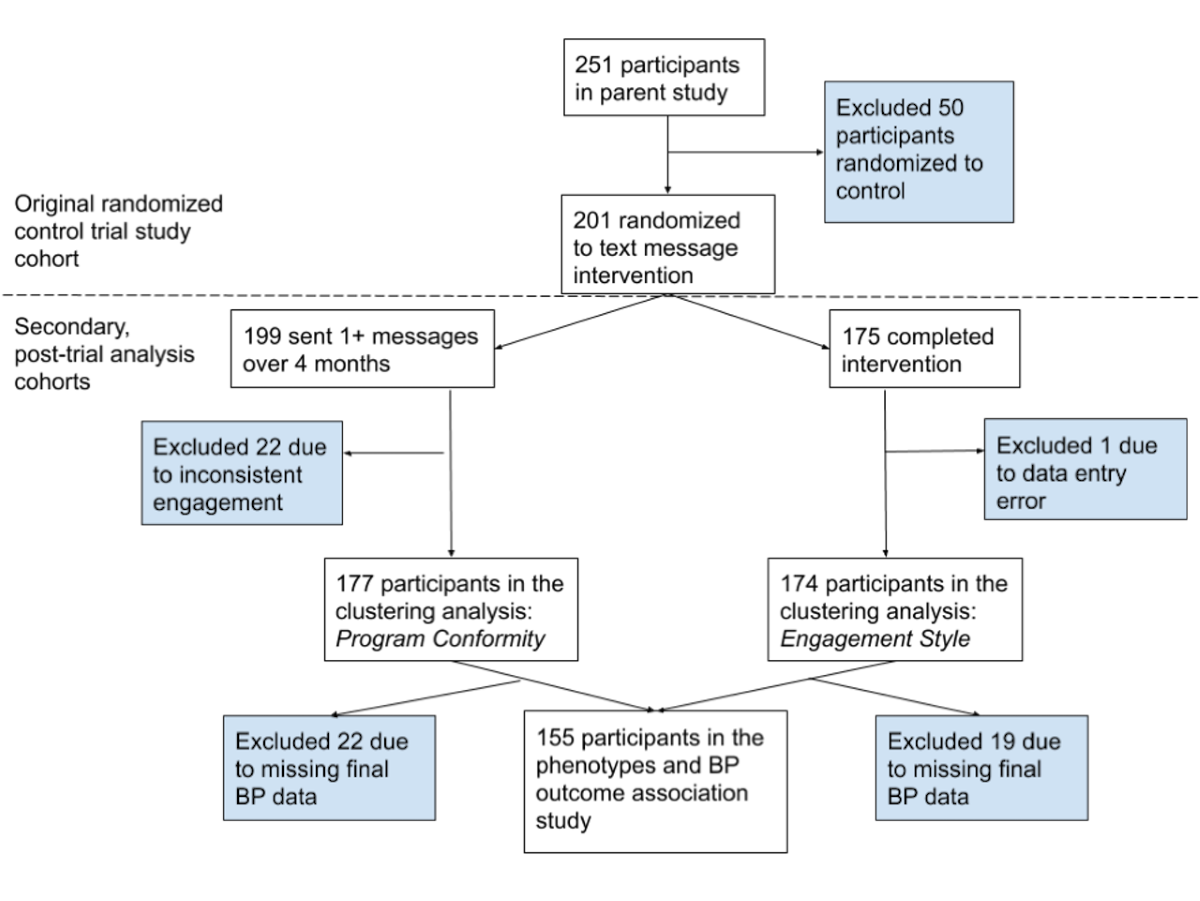
Flow diagram of participants included in each analysis.

In the original study, final BP was measured at an end-of-study visit. Three BP measurements were taken, and the average of the last 2 measurements represented the final reading. Cutoffs for goal BPs (uncontrolled BP) were in accordance with the American Heart Association/American Stroke Association Eighth Report of the Joint National Committee [20,21]. To identify associations between the interaction phenotypes and target BP, we applied the chi-square test for each cluster and dichotomous BP outcome (controlled vs uncontrolled). Only patients who had all 3 BPs successfully measured and entered into the database were included in the analysis. An overview of participant data subsets from the original study data included for each analysis is shown in Figure 2.

## Results

### Study Population and Data Characteristics

Of the patients who received automated text message reminders, the average age was 50.9 (SD 11.4) years and most were female (142/201, 70.6%; Table 1). From this cohort, we observed 42,263 text message interactions between patients and the automated text messaging platform. Of these interactions, 70.5% (29,791/42,263) of text messages were sent by the automated text messaging platform and 29.5% (12,472/42,263) of text messages were sent by patients. The most messages sent by a single patient was 347 messages. A total of 3.9% (491/12,472) of inbound text messages triggered an error message, and 23.2% (2899/12,472) of all inbound messages were unprompted. A total of 13.9% (1734/12,472) of all messages contained supplemental textual information submitted to the automated text messaging platform.

**Table 1:** Patient baseline characteristics by intervention group for original randomized control trial sans the control group (n=201).

Characteristics		RM+SS^a^ (n=100)		RM^b^ (n=101)	*P* value
Gender, female, n (%)		67 (33.3)		75 (37.3)	.07
Age in years, mean (SD)		51.9 (12.5)		50.7 (10.1)	.45
**Race, n (%)**		—^c^		—	<.001
	Black	86 (42.8)		95 (47.3)	—
	White	9 (4.5)		3 (1.5)	—
	Other	1 (0.5)		5 (2.5)	—
	Unknown	4 (2.0)		0 (0)	—
**Ethnicity, n (%)**		—		—	.96
	Hispanic or Latino	0 (0)		1 (0.5)	—
	non-Hispanic or Latino	99 (49.3)		100 (49.8)	—
	Unknown	1 (0.5)		0 (0)	—
**Insurance, n (%)**		—		—	<.001
	Private	42 (20.9)		56 (27.9)	—
	Medicaid	23 (11.4)		28 (13.9)	—
	Medicare	33 (16.4)		15 (7.5)	—
	None	1 (0.5)		2 (1.0)	—
	Unknown	1 (0.5)		0 (0)	—
Texts per patient user, m (SD)		66.9 (27.3)		57.3 (23.3)	.005
Active rate (patient sent at least one message), m (SD) days		139.5 (20.0)		138.3 (15.8)	.54
Processed responses–correctly formatted (BP^d^), mean (SD)		34.4 (11.4)		32.3 (13.0)	.14
Unprocessed responses–error message triggered (BP), mean (SD)		0.8 (1.3)		0.5 (0.7)	.02
Processed messages–medication adherence, mean (SD)		13.0 (4.6)		12.2 (4.9)	.18
Unprocessed messages–medication adherence, mean (SD)		0.9 (1.7)		0.8 (1.3)	.12
**Textual patterns for unprocessed messages, mean (SD)**					
	Character count (per message)	6.7 (10.2)		5.8 (7.0)	.65
	Token count (per message)	1.7 (2.7)		1.4 (1.9)	.92
	Word count (per message)	1.6 (2.5)		1.3 (1.7)	.75
	Number count (per message)	3.7 (3.7)		3.7 (2.1)	.83
**Temporal pattern (time of day), mean (SD)**					
	Morning, per user	50.7 (27.4)		42.3 (24.0)	.02
	Afternoon, per user	9.8 (8.1)		8.7 (7.3)	.33
	Night, per user	7.2 (7.6)		6.8 (5.6)	.73
	Late night, per user	4.1 (3.9)		4.1 (3.6)	.92
**Frequency over time, mean (SD)**					
	Per day	16.9 (11.1)		14.1 (9.9)	<.001
	Per week	109.7 (73.6)		87.6 (65.9)	.08
	Per month	418.1 (338.6)		361.4 (291.7)	.62
**Frequency each month, mean (SD)**					
	First month, per user	19.7 (7.1)	17.5 (5.5)		.02
	Second month, per user	17.7 (7.8)	15.2 (6.3)		.02
	Third month, per user	16.5 (6.8)	14.5 (5.5)		.03
	Fourth month, per user	14.2 (7.0)	12.6 (5.3)		.08

^a^RM+SS: remote text messaging with social support.

^b^RM: remote text messaging without social support.

^c^Not applicable.

^d^BP: blood pressure.

### Characterizing Interaction Phenotypes

#### Program Conformity

**Figure 3:**
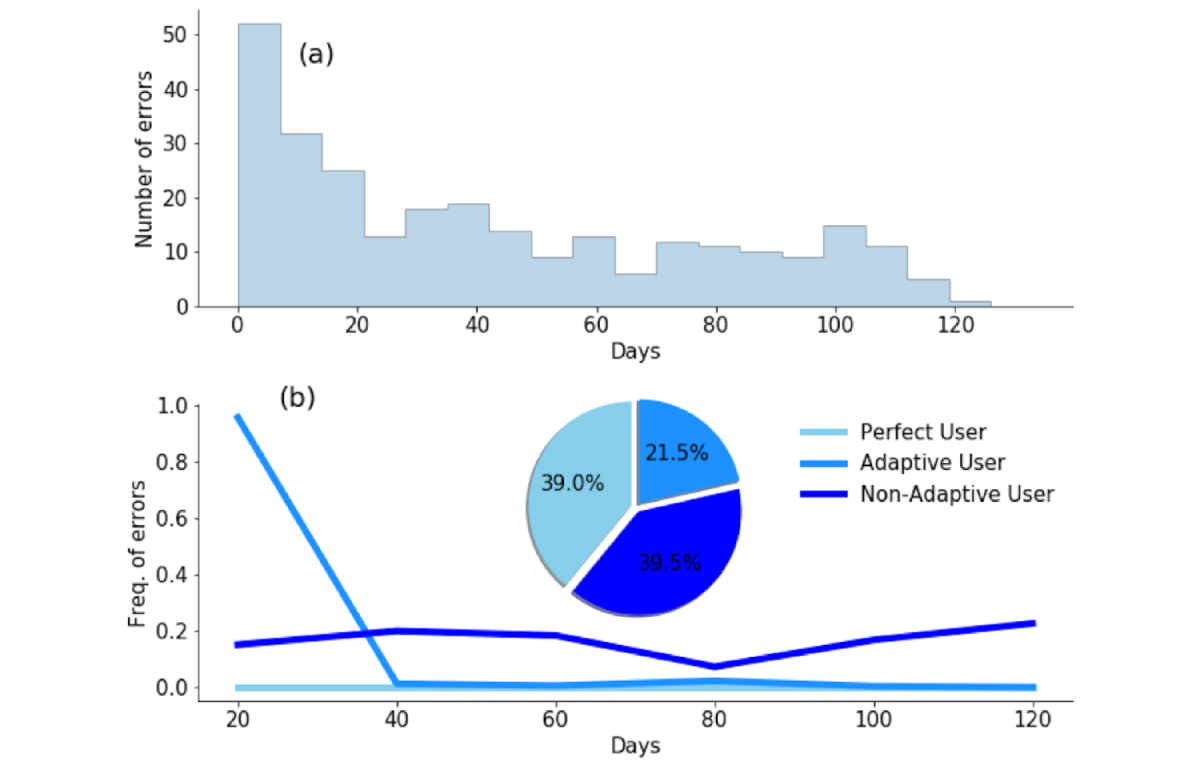
Program conformity clusters: (a) number of unprocessed responses (errors) throughout the 4-month period and (b) frequency of the conformer patterns and trends of errors over time based on program conformity user category.

For the 177 patients whose data were analyzed for program conformity, we observed a progressive decline in the number of errors generated by users over the 4 months of their study (Figure 3a). Most errors occurred within the first month overall. Using the *k*-means clustering algorithm, we identified 3 categories of patient program conformity with the text messaging platform defined by the frequency of error messages sent to the patient (Figure 3b). Almost 40% (69/177, 39.0%) of users did not receive any error messages (perfect users); 21.5% (38/177) received error messages within the first month and corrected their submissions for the remainder of the program (adaptive users); and 39.5% (70/177) consistently made errors over the course of all 4 study months (nonadaptive users).

#### Engagement Style

**Figure 4:**
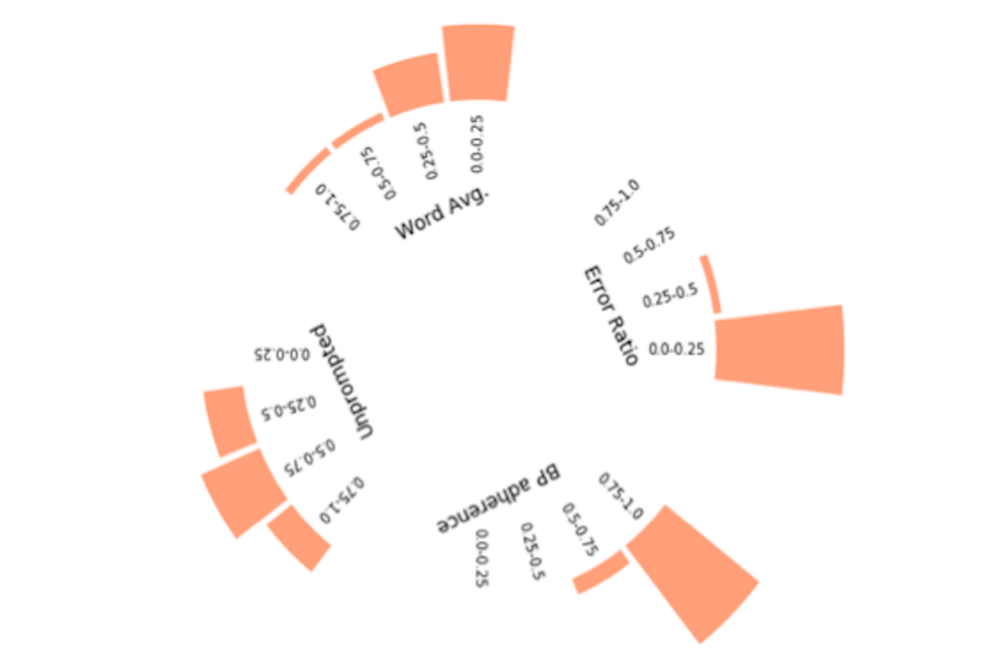
Engagement style: enthusiast—radial distribution of feature values.

**Figure 5:**
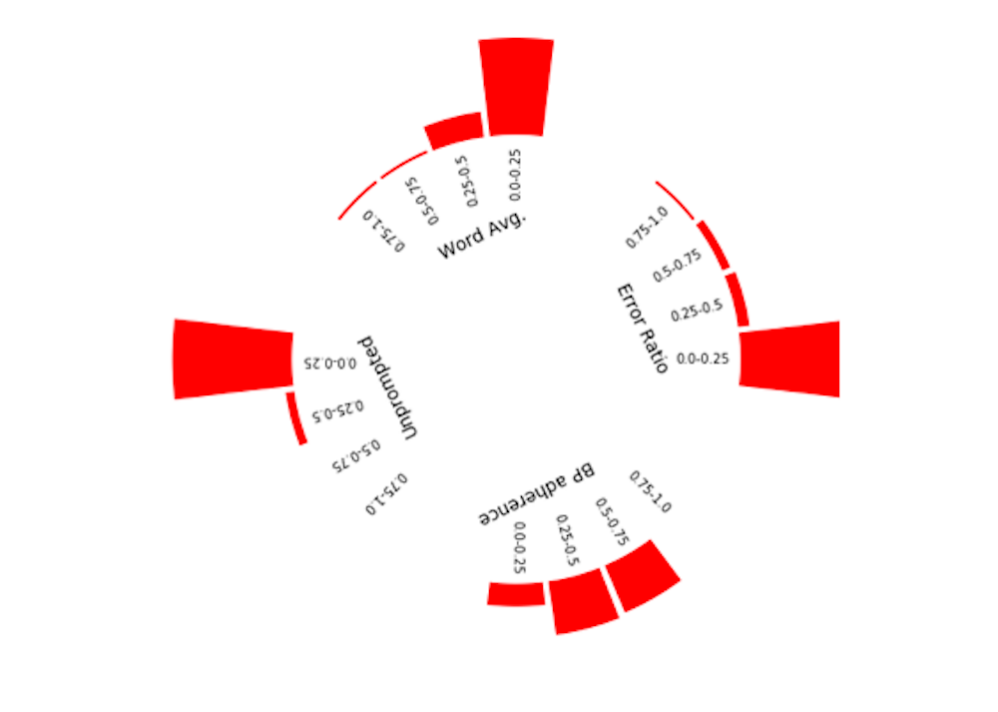
Engagement style: student—radial distribution of feature values.

**Figure 6:**
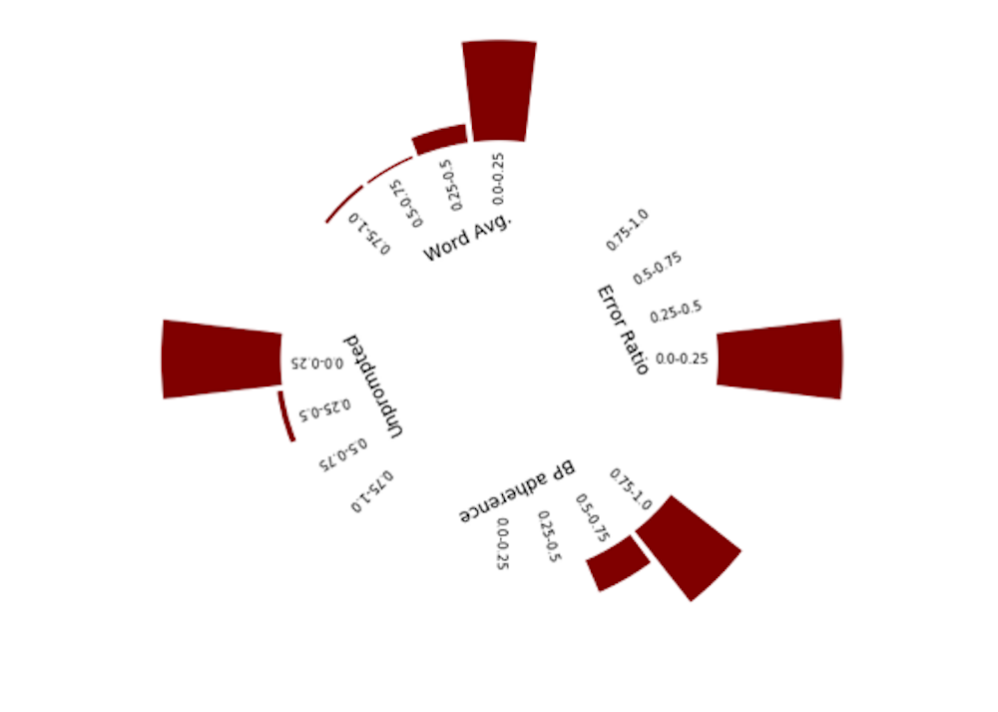
Engagement style: minimalist—radial distribution of feature values.

For the 174 users whose data were analyzed for engagement style, when we applied the *k*-means clustering method to engagement variables (scaled proportions of messages), we identified 3 notable categories of user engagement styles (Figures 4-6). For each cluster, we present the variables in a radial histogram accompanied by word lists where the size of the intent is associated with its use (Multimedia Appendices 1-3; larger = more frequent use of intent type by users in the cluster). The enthusiast (Figure 4) was characterized by high proportion of BP reporting adherence (range 0.50-1.0), low proportions of errors (range 0.0-0.5), higher proportions of unprompted messages (range 0.25-1.0), and mostly low but dispersed word averages (range 0.0-1.0). Most texts communicated pleasantries (“thank you”), BP with additional text (eg, “today it was,” “my bp”), and yes responses (eg, “ok” or “yes, I took it”). Notably absent intents included reports of feeling sick and appointment requests. The student (Figure 5) was characterized by low proportions and high dispersion of BP adherence (range 0.0-0.75), mostly low proportion but dispersed error rates (range 0.0-1.0), low proportion of unprompted messages (range 0.0-0.5), and low proportion but dispersed word averages. Most texts communicated BP, confirmation responses (eg, “yes” or “correct” from users), and communications about their medication(s). Reports about challenging life events were uniquely observed among this cluster (eg, “I’m having financial problems”). The minimalist (Figure 6) was characterized by higher proportions of BP adherence (range 0.5-1.0), low proportion of errors (range 0.0-0.25), low proportion of unprompted messages (range 0.0-0.25), and low and dispersed word averages. Like the enthusiast, the minimalist texts communicated mostly pleasantries, BP with additional text, and yes responses. Additional unique intents observed include compensation and requests to adjust reminders.

Table 2 further describes engagement style clusters by patient characteristics (race, sex, age) and study arm. For all engagement styles, we observed similar distributions of sex and age. Black was the majority race across all clusters. Most enthusiasts were assigned to the arm with social support. Although most messages conveyed one intent, a notable proportion of messages conveyed two intents across all clusters. More complex messages with multiple intents were infrequent.

**Table 2:** Distribution of participant characteristics by engagement style.

Characteristic		Enthusiast (n=17)	Student (n=45)	Minimalist (n=112)
Gender, female, n (%)		12 (71)	25 (56)	82 (73)
Age in years, mean (SD)		57.9 (7.3)	47.3 (11.8)	52.4 (11.1)
**Race, n (%)**				
	Black	17 (100)	40 (89)	99 (88)
	White	0 (0)	1 (2)	8 (7)
	Other	0 (0)	3 (7)	2 (2)
	Unknown	0 (0)	1 (2)	3 (3)
**Study arm, n (%)**				
	Remote monitoring	4 (24)	26 (58)	57 (51)
	Remote monitoring + social support	13 (77)	19 (42)	55 (49)
**Total messages submitted, n (%)**				
	1 intent (%)	586 (83)	345 (87)	694 (86)
	2 intents (%)	114 (16)	43 (11)	103 (13)
	3 intents (%)	5 (1)	5 (1)	8 (1)
	4+ intents (%)	2 (1)	2 (1)	1 (1)

### Identifying Associations Between Interaction Phenotypes With Blood Pressure Outcomes

We characterized the relationship between the 6 phenotypes and BP outcome, controlled versus uncontrolled, for the patients who completed the program and had all 3 end of study visit BPs successfully measured (Table 3). We observed no statistically significant differences among the program conformity clusters. However, among engagement style clusters, a greater proportion of patients in the minimalist cluster achieved controlled BP phenotype (*P*<.001).

**Table 3:** Association between interaction phenotypes, program conformity, and engagement style, with BP outcomes (n=155).

Interaction phenotype type		Total, n (%)	Users achieving target BP^a^, n (%)	*P* value
**Program conformity**				
	Perfect user	51 (32.9)	31 (60.7)	.12
	Adaptive user	66 (42.6)	39 (59.1)	.14
	Nonadaptive user	38 (24.5)	23 (60.5)	.19
**Engagement style**				
	Enthusiast	17 (11.0)	7 (41.2)	.47
	Student	35 (22.6)	19 (54.3)	.61
	Minimalist	103 (66.5)	67 (65.0)	<.001

^a^BP: blood pressure.

## Discussion

### Principal Findings

We identified distinct patterns of patient interaction phenotypes with automated text messages for hypertension management including program conformity and engagement style. We assessed whether these distinct patterns of interaction phenotypes were associated with achieved BP control.

### Program Conformity

We first explored heterogeneity in adhering to the structured interactions requested by the automated text messaging platform, using the system’s rate of error messages returned to the patient submission as a proxy for program conformity. Perfect users adopted the rules at the beginning of the trial, while adaptive users did so within a month. Nonadaptive users continued to generate error messages throughout the course of the 4-month program.

Our analysis suggests that many patients do not change behavior despite repeated reminders, as the rate of error messages did not change throughout the study’s 4-month duration for nonadaptive users. This observation is significant because automated text interventions for hypertension and many other clinical applications predominantly use restricted and structured interactions by limiting patient communication to discrete submissions in a prespecified, standardized format. In our case, there were also time windows for submission so that only a single piece of information (ie, BP or medication adherence) was being collected at a time. This simplified approach makes automated texting technically feasible and approachable across many clinical settings [17,22-24]. Because texting is a largely unrestricted platform, automating a response to correct unstandardized submissions is a way to train users to conform with the program.

However, for even trained users, less restrictive texting or additional means for communication might be desired. A total of 13.9% of messages contained additional text data submitted to the automated texting platform. These messages could have prompted additional engagement between the text messaging platform and the patient or stimulated a conversation between a health care provider and patient. Those implementing automated text systems through structured interactions should bear in mind that a portion of users may never completely adapt to a limited, structured format of response and reply communication. Accounting for heterogeneity in engagement patterns may be one important way to acknowledge the complexity of health-related behaviors [25]. It is likely also the starting point for an equitable approach to automated communication, as those with disabilities or low or limited literacy are more likely to report difficulty texting or following instructions [26]. Automated texting that leads to conversation may result in better data collection, patient engagement, and clinical outcomes.

### Engagement Style

Heterogeneity in mobile health engagement styles may explain why such interventions have only demonstrated modest to equivocal clinical impact [27]. In our evaluation, only the minimalist engagement phenotype was associated with significantly better BP control. The patients in this cluster seemed to have a straightforward, business-like relationship with the program, with tight adherence to the original design of the platform. This suggests that for some patients, automated texting with limited structured interaction is sufficient to achieve target BP. The implication is that, while evidence generally suggests text-based interventions should be supplemented by additional care components [28,29], some patients will do just fine with the minimal version. Alternatively, these patients may have achieved target even without any intervention, although all patients were above target at enrollment.

Identifying strategies tailored to other phenotypes may result in better clinical outcomes. The enthusiast demonstrated high levels of engagement, regularly sending BP readings in the appropriate format, as suggested by their low error rates. They also tended to submit more unprompted messages, and compared with other clusters, were hyperverbal, often relaying pleasantries. Despite their high engagement, this phenotype was not associated with significantly improved target BP, although the calculation may have been underpowered. This discrepancy merits further evaluations, and future interventions might consider conversational approaches for this phenotype to improve clinical outcomes.

The student cluster demonstrated more variable BP reporting adherence and error rates, suggesting they faced some challenges in abiding by the rules of the program. Based on their message intents, the students sought more guidance, asked more questions, and had more complex needs. For this group of patients, structured automated programs for BP monitoring may be insufficient to meet all their needs. Importantly, they also relayed clinically significant and meaningful information that was lost and unrecognized by a structured, automated platform. It is possible that the structured format even deterred engagement. In a study of text message communication for mental health among black women, participants commonly cited an impersonal feel and inadequate communication as barriers to adoption [30], and restrictive automated communication may contribute to similar sentiments in this cohort. In comparison with other communication style clusters, the student group tended to skew older and were randomized more frequently without the social support group, which may have affected their behavior. We observed that patients in the RM+SS arm submitted more messages and were more verbose, which could suggest that social support influenced the degree of patient engagement with the platform.

### Implications for Next Steps

Our observations have important implications for designing future text messaging and hypertension interventions. Evidence suggests that the most effective interventions have multiple strategies for addressing the informational, behavioral, and social barriers to health and are more effective in larger doses (eg, more time) over a longer period of time [31]. Examples of such strategies include patient education sessions, case management, group support meetings, rewards for meeting BP goals, and pillboxes or medication reminders. However, such complex interventions may also be more resource intensive, and our results suggest that for some, the marginal cost may be unnecessary. Rather than use one universally complex and potentially costly approach, more sustainable interventions might start with automated text support for hypertension management. Additional resources could then be targeted toward those who remain difficult to engage or whose BPs remain uncontrolled; some may require intensive in-person care. Further, our results suggest that identifying patients who send texts outside of the structured format or time windows may present opportunities to engage with patients and identify additional resources to support their care.

### Strengths and Limitations

This study has several strengths. To our knowledge, this is the first study to examine variations in the way patients engage with automated text. We leveraged data from a randomized trial and therefore were able to access a large text data file. This analysis included both quantitative and qualitative components, which was possible because we qualitatively coded all text messages.

There are some limitations. This was an exploratory analysis of data from a single center study. No conclusions can be drawn about the specific makeup of clusters in other settings. Clusters defined using another *k* could identify other phenotype patterns and correlate with BP outcomes, although our sample size limited exploration of greater numbers of *k* clusters.

Another limitation is that due to incomplete datasets, each of the 3 analyses presented here were conducted in different samples of the study population (n=177, n=174, and n=155, respectively). The sample size was smallest for the analysis of association between text phenotype and BP outcomes because we conducted a complete case analysis. Due to the large number of missing end-of-study BP measurements, imputation of missing values was not justified. Analysis of associations between BP outcomes with behavioral phenotypes was therefore likely limited by small sample size. However, we had a large volume of text messages for our analytic datafile. Also, although the program was designed to be completely automated, on rare occasions it was used to communicate with patients in real time via text regarding study-specific logistics such as study follow-up appointments and reimbursement. This likely altered engagement dynamics for a small number of patients. In addition, the phenotypes described are only pertinent for a short period, as the study was 16 weeks in duration.

### Conclusion

Automated texting using a limited, structured interaction format is likely effective in improving BP control for a unique patient phenotype. For others, this format is likely inadequate, and more comprehensive communication and needs assessment may be required. How quickly patients adapt to automation may be less important than how they engage. In particular, patient engagement outside of structured text interactions may signal the need for additional intervention. Future research should identify unique patient phenotypes so that interventions can be tailored accordingly. More research is needed to understand, design, and enhance automated texting platforms so all patients, regardless of phenotype, can reach their BP goals.

## Appendix

Multimedia Appendix 1 Enthusiast phenotype word cloud.

Multimedia Appendix 2 Minimalist phenotype word cloud.

Multimedia Appendix 3 Student phenotype word cloud.

## Abbreviations

BP: blood pressure
RM: remote monitoring only group
RM+SS: remote monitoring + social support group
