# Supplementary figures and images for: Patient Interaction Phenotypes With an Automated Remote Hypertension Monitoring Program and Their Association With Blood Pressure Control: Observational Study
Source: J Med Internet Res. 2020 Dec 3;22(12):e22493. doi: 10.2196/22493 (PMC7746494; doi:10.2196/22493)

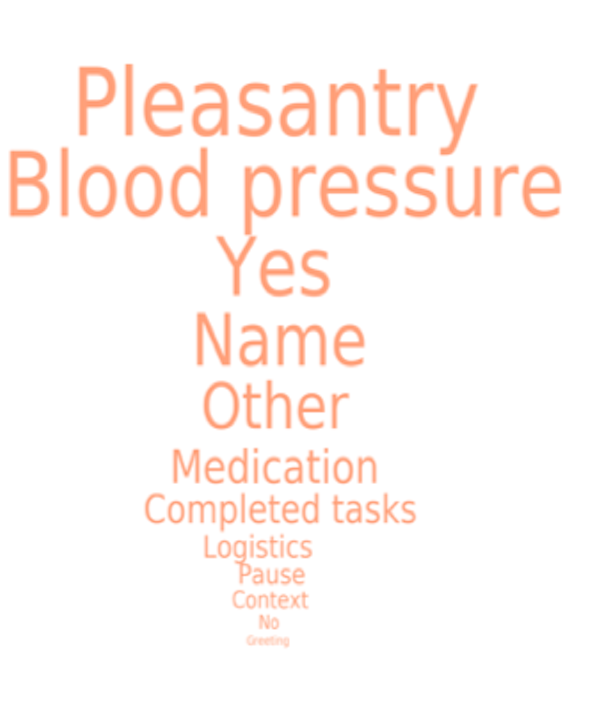

Supplement: Multimedia Appendix 1 [file jmir_v22i12e22493_app1.png]

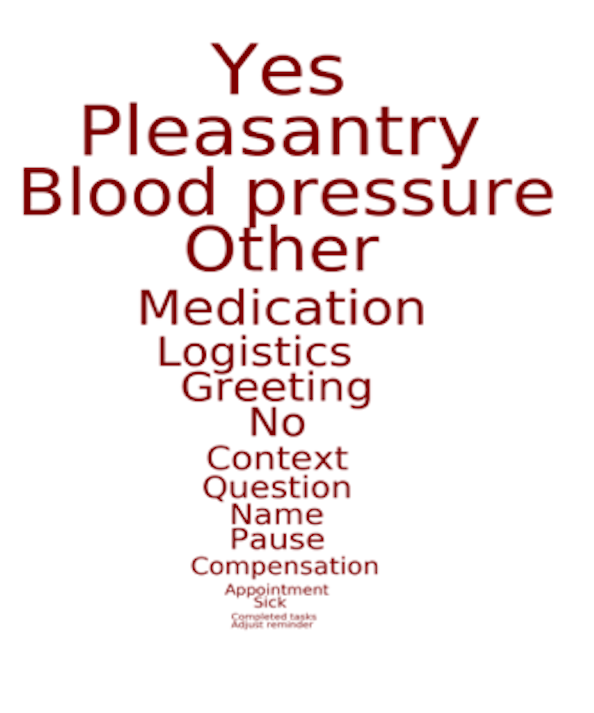

Supplement: Multimedia Appendix 2 [file jmir_v22i12e22493_app2.png]

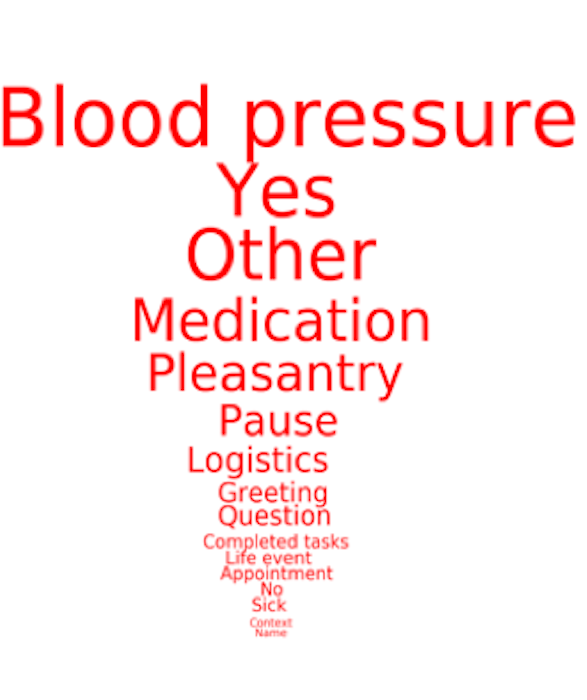

Supplement: Multimedia Appendix 3 [file jmir_v22i12e22493_app3.png]
